# High return to sports rates after operative treatment of patella fractures

**DOI:** 10.1186/s40001-023-01359-1

**Published:** 2023-09-22

**Authors:** Sebastian Pesch, Frederik Greve, Michael Zyskowski, Michael Müller, Moritz Crönlein, Peter Biberthaler, Chlodwig Kirchhoff, Markus Wurm

**Affiliations:** 1 Department of Trauma Surgery, Klinikum Rechts Der Isar, Technical University of Munich, Ismaninger Strasse 22, 81675 Munich, Germany; 2 Department of Trauma Surgery, AUVA Trauma Center Meidling, Kundratstrasse 37, 1120 Vienna, Austria

**Keywords:** Patella, Fracture, ORIF, Return to sports, Functional outcome

## Abstract

**Background:**

Patella fractures are relatively rare fractures and only little is known about the postoperative return to sports after patella fractures.

**Methods:**

This retrospective study presents information on functional outcome after operative treatment of patella fractures as well as time until return to sports and patients’ complaints after open-reduction internal-fixation (ORIF) of patella fractures.

**Results:**

Overall, 39 patients after ORIF of patella fractures were evaluated at our Level-I trauma center with a mean follow-up of 42 months. The mean time until return to sports was 7 ± 3.9 months. No significant difference was found for functional outcome with respect to body mass index (BMI) or age. Fracture consolidation was accomplished after a mean of 6.9 ± 2.9 months besides a relatively low complication rate of 5.1% (*n* = 2).

**Conclusion:**

The results demonstrate a high return to sports rate of 90.3%. However, only 51.6% were able to perform sports on their pre-injury level or above.

*Trial Registration* The study was retrospectively registered at DRKS (No: DRKS00031146).

## Background

The incidence of patella fractures (< 1%) is relatively low compared to other fracture entities and mainly occurs in patients aged between 20 and 50 years [1, 2]. To date, various operative treatment options are available in the literature ranging from minimally invasive surgical procedures to open techniques [2, 3]. The key points for successful management are the integrity of the extensor mechanism, articular congruity and no displacement of fracture fragments [4, 5].

The choice of surgical treatment depends on patients’ characteristics and the fracture pattern [6]. Complex fracture patterns should undergo a computed tomography (CT) scan for preoperative planning [7–9]. Surgical treatment options include a wide range of tension-band wiring techniques, screw fixation and angle-stable plate fixation [6, 9]. In 2019, a survey of current surgical treatment standards for patella fractures showed that tension-band wiring still is the preferred surgical approach (30%) [7, 8].

The latest research results postulate better clinical outcome in angle-stable plate fixation with lower complication rates, especially in comminuted fractures with osteopenia/osteoporosis [10, 11]. Regarding the functional outcome after surgical treatment of patella fracture, literature provides heterogeneous results [12, 13] and little is known about the return to sports (RTS) and postoperative complaints. Functional outcome does not significantly improve after implant removal of tension-band reconstruction with cannulated screws or k-wires, respectively [14]. To the best of our knowledge, to date, there is no study addressing the return to sports after open-reduction-internal-fixation (ORIF) of patella fractures.

One aim of this study was to gather information with respect to RTS as well as complaints during sporting activity and especially the influence of fracture patterns, age and BMI compared to the functional outcome. The hypothesis of this study was that patients show high-to-excellent results after ORIF of patella fractures.

## Methods

Between 2004 and 2016, all consecutive isolated patella fractures which were operatively treated in our Level-I trauma center were prospectively enrolled in this study. Exclusion criteria of this study were: patients’ age below 18 years, pregnancy, delinquent patients, polytraumatized patients (Injury Severity Score (ISS) > 16), (intraarticular) lesions/fractures of the ipsilateral leg, ipsilateral (patellofemoral) osteoarthritis, conservative treatment, missing information. The used operative techniques were: tension band wiring, single/multiple screw osteosynthesis and angle-stable plate fixation.

Patients were asked to participate in all routine follow-up (FU) examinations after 6, 12, 26 and 52 weeks of surgery. Patients received clinical as well as radiologic assessment at all routine follow-up examination appointments. Fracture consolidation was evaluated by two independent reviewers by X-ray (Consultant of the Department of Radiology and Consultant of the Department of Trauma Surgery). Computed tomography was only performed in case of clinic evident non-union. Furthermore, all patients completed the Munich Knee Questionnaire (MKQ) and the sports activity questionnaire. Patella fractures were classified according to the AO/OTA Classification system.

Functional outcome as well as the return to sports was assessed at a minimum of 6 months postoperatively using the Munich Knee Questionnaire (MKQ) [15]. The RTS questionnaire was evaluated at a minimum of 6 months postoperatively and after radiologic assured fracture consolidation.

The RTS assessment comprises information about body mass index, sports activity (recreational, competitive or professional level), specific complaints during training (anterior knee pain (AKP), joint swelling, numerical rating scale (NRS)), time until return to sports as well as alteration of sports level.

Data is provided as arithmetic mean and standard deviation. Statistical analysis was performed using the SSPS software (IBM Corp. Released 2013. IBM SPSS Statistics for Macintosh, Version 22.0. Armonk, NY: IBM Corp.). All statistical tests were performed two-sided and a level of significance (α) of 0.05 was set for all tests.

The study was approved by the Institutional Review Board (Technical University Munich, 409/15s).

### Operative technique and postoperative management

**Fig. 1:**
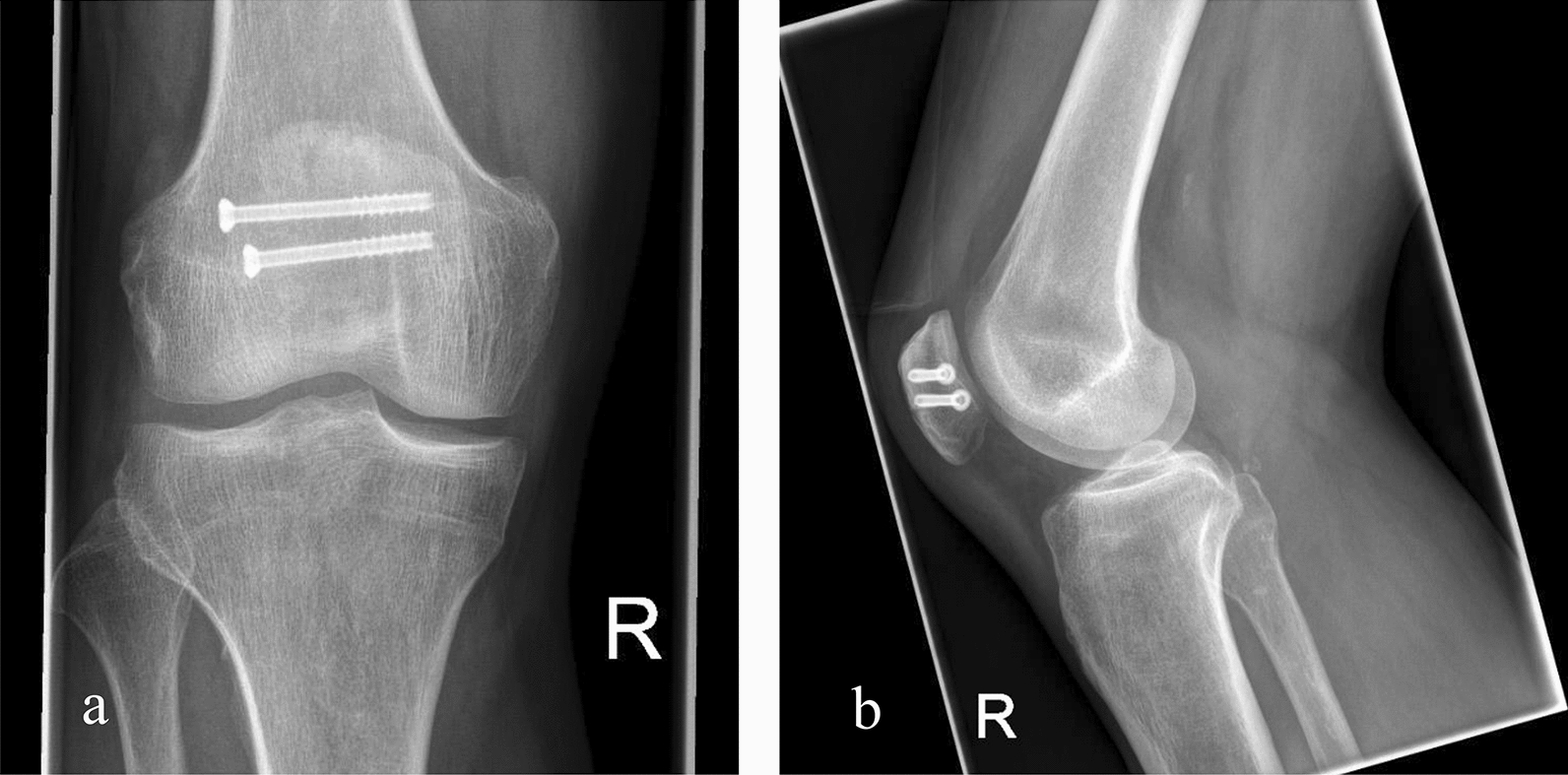
a/p (**a**) and lateral (**b**) X-ray of a simple transverse patella fracture after operative treatment using screw fixation

**Fig. 2:**
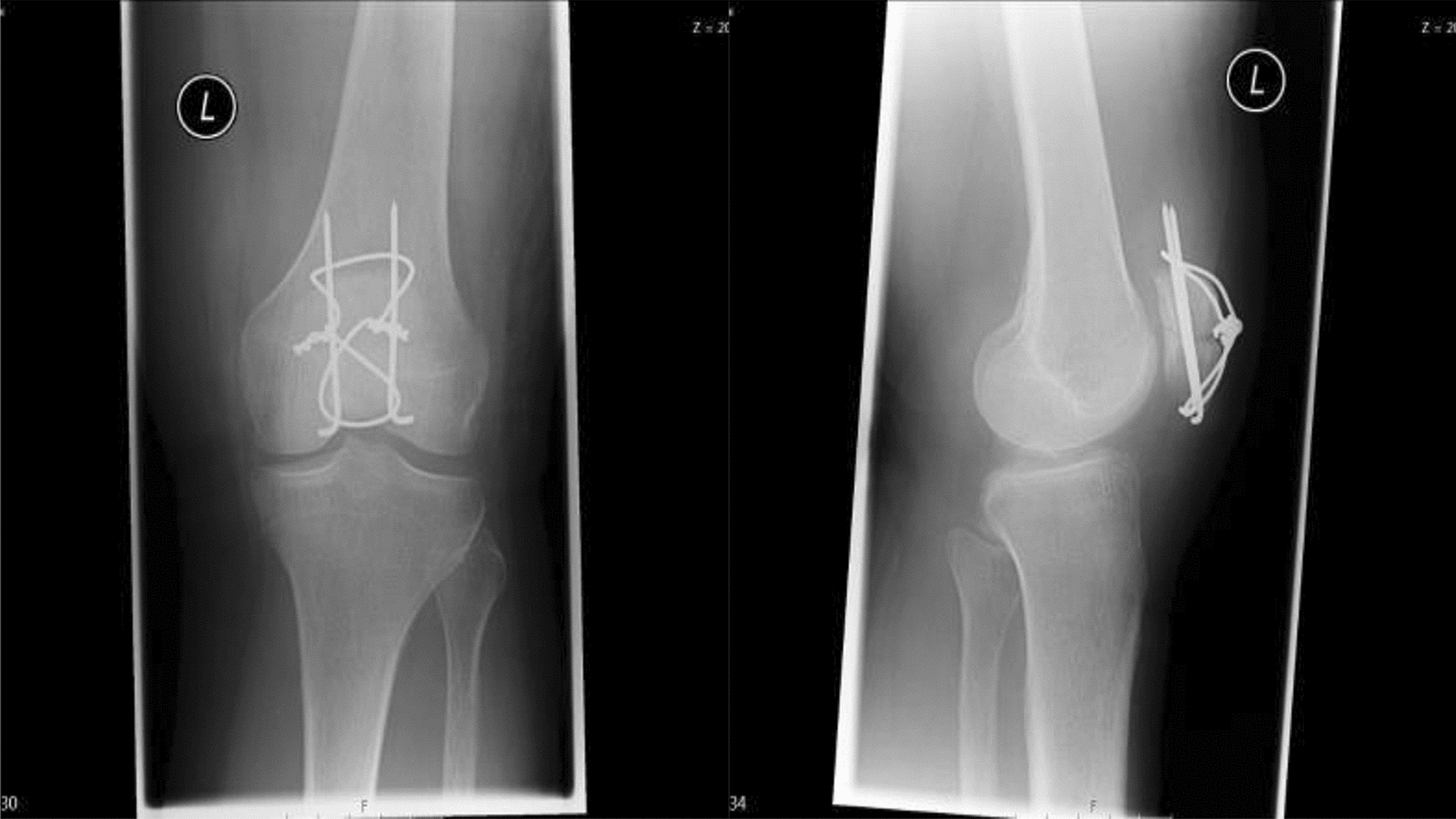
a/p (**c**) and lateral (**d**) X-ray of a transverse patella fracture after operative treatment using tension-band wiring

**Fig. 3:**
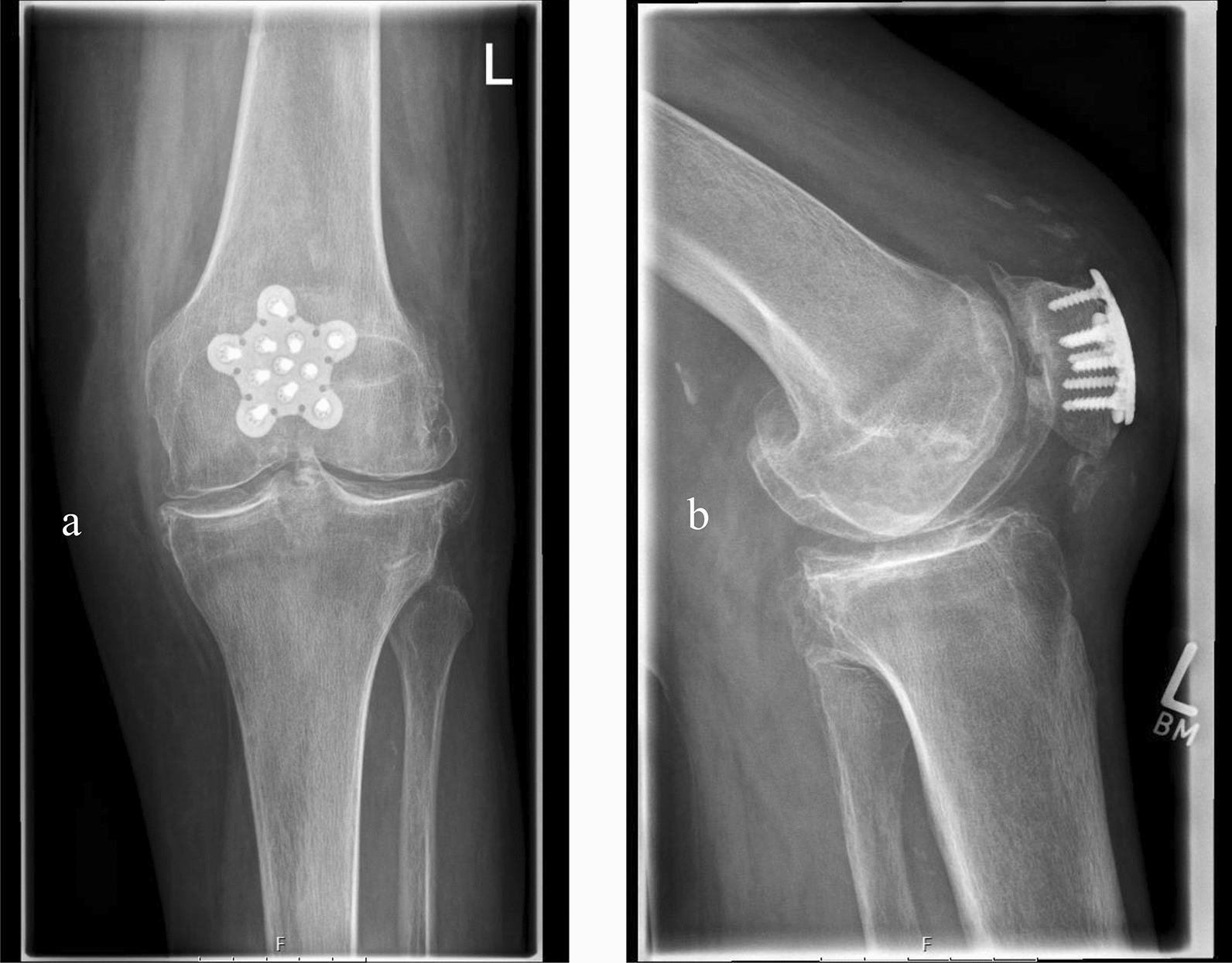
a/p (**a**) and lateral (**b**) X-ray of a multifragmentary patella fracture after operative treatment using a locking plate

Surgical treatment was adapted to the fracture pattern according to the “Orthopaedic Trauma Association” (OTA)—classification and soft tissue conditions. Patients were treated either using screw fixation (Fig. 1), tension-band wiring (Fig. 2) or locking plate osteosynthesis (Fig. 3). Simple transverse fractures were mostly treated using tension-band wiring or screw fixation. Comminuted fractures were treated using locking plate fixation. All surgical procedures in this study were open techniques.

The postoperative treatment included a gradually increased range of knee flexion of 30° every two weeks and half to full weight-bearing for six weeks depending on the fracture severity. Type A fractures with avulsion of the inferior pole were treated with half weight-bearing and gradually increased range of motion depending on surgical treatment and type B fractures with full weight-bearing on axial load. Type C fractures were treated according to surgeons’ preferences and depending on the intraoperative conditions. Rehabilitation is routinely performed via outpatient physiotherapy (2–3 times a week) depending on the severity of the fracture.

## Results

39 patients met the inclusion criteria and were operatively treated at our Level-I trauma center. The mean age was 56 ± 17 years (male patients *n* = 16, female patients *n* = 23) and the mean follow-up was 42 ± 34.2 months (range: 7–139 months). The mean body mass index was 24.7 ± 5.5. Surgical treatment with tension-band wiring (see Fig. 2) was conducted in 19 patients. Nine patients received screw fixation (see Fig. 1) and five patients were operated using both screw fixation and tension-band wiring. Locking plate fixation was done in six patients (see Fig. 3). Fracture consolidation was accomplished after a mean of 6.9 ± 2.9 months. No mal or non-unions were seen in the follow-up examinations in this cohort. According to the OTA classification, the following fracture types were ascertained: AO34-C1.1/2 (*n* = 14), AO34-C1.3 (*n* = 2), AO34-B2.1 (*n* = 4), AO34-C2 (*n* = 2), AO34-C3 (*n* = 2) and AO34-C3.2 (*n* = 15). No Type A fractures were enrolled in this cohort.

### Return to sports

79.4% (*n* = 31) of all patients performed sports prior to their patella fracture. 28/31 patients (90.3%) returned to at least one sporting activity in contrast to three (9.7%) patients without sports activity after at least 6 months of follow-up. Eight patients (*n* = 8) did not perform sports prior to their patella fracture and none of these patients started a sporting activity at the final follow-up examination at 1 year postoperatively.

“Recreational” sports was reported by 25 (80.6%) patients, “competitive” sports by six (19.4%) patients and no patient reported on a “professional” sports activity. The frequency of performing sports was reported once a week by eight (28.6%) patients, two to three times a week by 11 (39.3%) patients and more than three times a week by nine patients (32.1%).

16 (51.6%) patients reported on an equal sports level compared to the preoperative state. 11 (35.5%) patients reported on a decrease and one (3.2%) patient of an increased sports level.

Overall, 21 (67.7%) patients were satisfied with their postoperative sports level.

The mean time until RTS was 7 ± 3.9 months. Pain, rated using the NRS, was reported with a mean of 3 ± 1.9 during sports after at least 6 months of follow-up. The majority (96.4%) did not take any medication for pain relief at this time (*n* = 27).

Anterior Knee Pain (AKP) was reported by 13 (46.4%) patients as “never”, 12 (42.9%) patients “occasionally” and three (10.7%) patients on a “regular basis”. Pain on doing “jump squats” was reported by eight (28.6%) patients without other complains. Nine (32.1%) patients reported “occasionally” about complaints and 11 patients (39.3%) with complaints on a “regular basis”.

Besides AKP, 18 (64.3%) patients did not notice knee swelling after training, yet nine (32.1%) patients reported on knee joint swelling one a “occasional” basis and one (3.6%) patient on a “regular basis” (see Table 1).

**Table 1:** Descriptive statistics with respect to return to sports and postoperative complaints

Time of return to sports	7 ± 3.9 months
Patients return to sports	28 (90.3%)
Recreational sports	25 (80.6%)
Competitive sports	6 (19.4%)
Professional sports	0
Activity (1x/w)	8 (28.6%)
Activity (2-3x/w)	11 (39.3%)
Activity (> × 3/w)	9 (32.1%)
Even sports level prior to surgery	15 (48.4%)
Increased sports level	1 (3.2%)
Decreased sports level	12 (38.7%)
Satisfaction with postop sports level	21 (67.7%)
Pain using numeric rating scale	3 ± 1.9
Medication (such as NSAID)	1 (3.1%)
Anterior knee pain	
Never	13 (46.4%)
Occasionally	12 (42.9%)
Regular	3 (10.7%)
Pain during jump squat	
Never	8 (28.6%)
Occasionally	9 (32.1%)
Regular basis	11 (39.3%)
Knee swelling	
Never	18 (64.3%)
Occasionally	9 (32.1%)
Regular basis	1 (3.6%)

### Functional outcome

Functional outcome measured using the MKQ showed a mean of 65.3% ± 17.4 which represents a good result. The mean range of motion of the affected knee was 89.1° ± 17.4° (range: 60–120°) 6 months postoperatively. There was no statistically significant difference in functional outcome in patients’ age under or above 50 years (*p* = 0.4). Furthermore, no significant difference was found in patients with a BMI > 25 (*p* = 0.6) compared to a BMI < 25. The mean BMI was 24.7 ± 5.5 (range 16–46). No significant impact was evident for patients with a RTS later than 12 months (*p* = 0.6) compared to patients who performed sports on an earlier basis after ORIF (Table 2).

**Table 2:** Postoperative functional outcome 6 months postoperatively

Functional outcome	
Range of motion	89.1° ± 17.4°
Munich Knee Questionnaire	65.1 ± 17.4
Patients’ age below 50 years	67.5 (*n* = 15), *ns
Mean patients’ age (range)	56 ± 17 (range: 25–81)
Body mass index below 25	66.5 (*n* = 24), *ns
Mean body mass index (range)	24.7 ± 5.5 (16–46)
Return to sports after 12 months	69.9 (*n* = 17), *ns

**Fig. 4:**
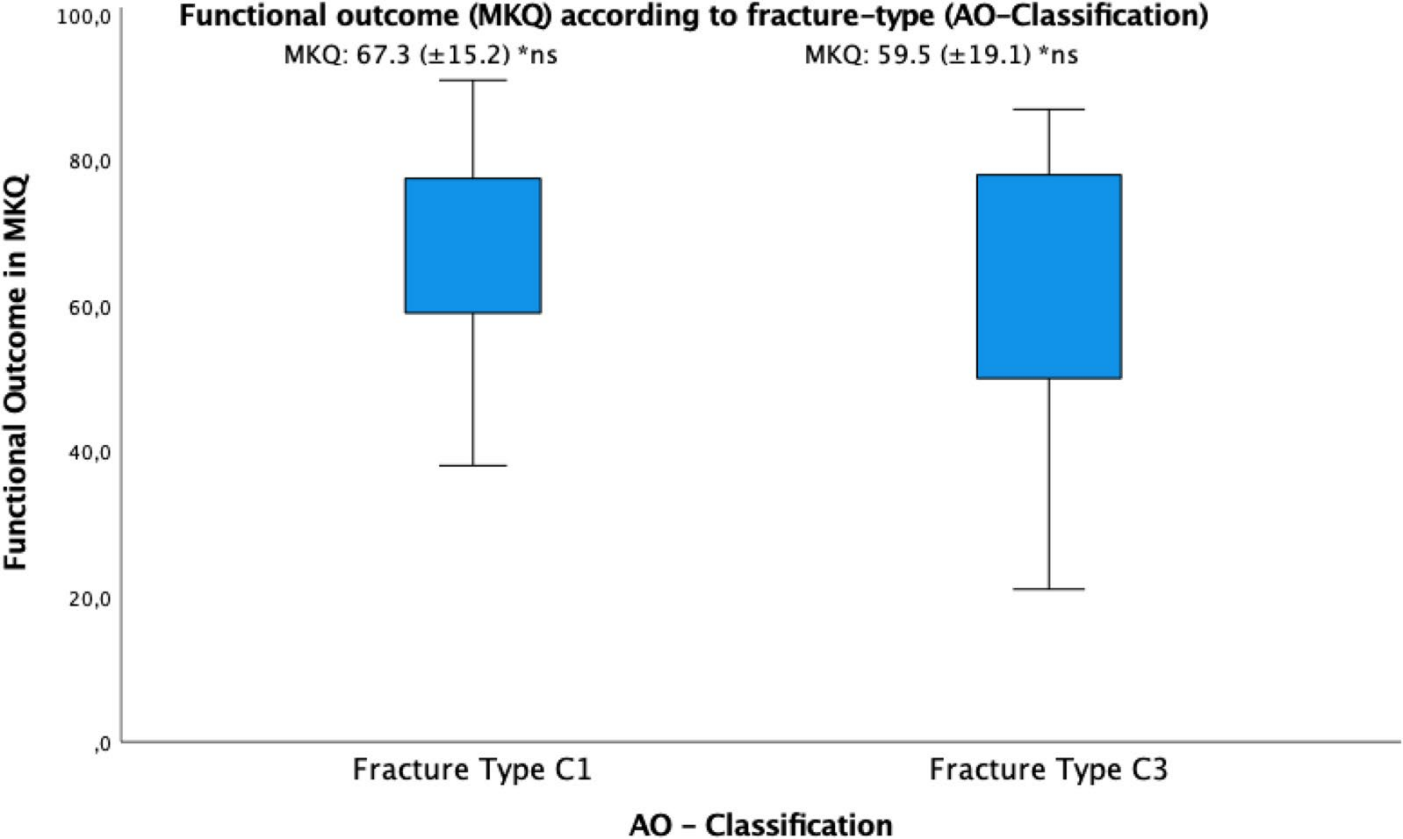
Box plot of functional outcomes (MKQ) based on the fracture type (AO classification)

Regarding the fracture severity, there was a less favorable functional outcome in AO-C3-type fractures MKQ: 59.5% ± 19.1, yet without statistically significant results compared to AO-C1 fractures (MKQ: 67.3% ± 15.2) (*p* = 0.2) (Fig. 4). Furthermore, a less favorable functional outcome was seen in patients without sports activity after treatment (MKQ: 56.9% ± 21.2), however without a significant difference to patients with sports activity (MKQ: 69.2% ± 15.6) (*p* = 0.1). Comparing the functional outcome in patients with pre-injury sports activity (MKQ: 66.0% ± 18.2) to patients without performing sports prior to their injury (MKQ: 64.8% ± 18.3), no significant difference was evident either (*p* = 0.9).

### Complications

The overall complication rate (5.1%) was relatively low. One patient suffered from implant failure within 6 weeks after a figure-of-eight wiring and revision surgery was carried out due to loss of reduction. Furthermore, one patient who was initially treated using screw fixation which showed a refracture within 8 weeks after ORIF and had to be revised using plate fixation.

## Discussion

28 of 31 (90.3%) patients returned to at least one sporting activity after a minimum of at least 6 months of follow-up. However, the preoperative sports level was only reached by 16 patients (51.6%). This information adds to the current literature since to the best of our knowledge there is no study which reaches out to answer these questions.

Another important finding was the mean time until RTS of 7 ± 3.9 months, which is comparable to other lesions around the patella in the current literature [16, 17]. In 2020, Beranger et al. reported on high return to sports rates (94.4%) after patellar tendon ruptures [18]. Overall, 83% of patients reached their pre-injury sports level with a prolonged time of recovery of 17 months. Compared to our results, a slightly lower RTS rate was seen (90.3%), yet patients returned to sports earlier after a mean of 7 month. A systematic review from Haskel et al. in 2021 reported on high RTS rates (88.9%) including high return to pre-injury sports levels (80.8%) after ruptures of the extensor tendons [19]. These findings go along with results from Patel et al. in 2022 who reported on a 71% return to play rate in 21 professional soccer players after a mean of 7 months after patellar fracture which corresponds to our findings [20]. A systematic review by Grondin et al. reported on 196 patients suffering from patellar tendon ruptures and a return to sports between 52 and 100% which is also comparable to our findings [21].

Not all of these findings reported on the preoperative sports level why no comparison to our results can be made. From the authors point of view the measurement of preoperative and postoperative sports level is important for the patients since the decrease from competitive to recreational can be cumbersome for semi-professional athletes.

The higher rate of return to the pre-injury level (83 and 80.8%) compared to our group of patients (51.6%) could be explained by the different mean age of the study groups and general differences when comparing “soft tissue” injuries of the knee to fractures also affecting the extensor mechanism. However, 75% of presented patients (*n* = 21) were satisfied with their post-ORIF sports level. 15 patients did reach their preoperative sports levels and one patient even increased his sportive activity from recreational to competitive. In contrast, 12 patients decreased their sports level after ORIF of their patella fracture. Some of these patients may have increased their sportive activity over time which can could have potentially biased the results.

However, the comparison of soft tissue injuries to fractures are debatable. In general soft tissue injuries around the knee revealed higher sports levels (i.e., quadriceps tendon ruptures, patella tendon ruptures, medial patellofemoral ligament (MPFL) ruptures) compared to our results after patella fractures [17, 19]. The RTS questionnaire included activity level (recreational, competitive or professional level), complaints during sports (anterior knee pain, knee joint swelling and numerical rating scale) and the return to patients’ pre-injury sports level. None of the presented patients was a professional athlete yet six patients (19.4%) reported on a competitive sports level prior to their patella fracture. 21 (67.7%) patients were satisfied with their postoperative sports level yet not all of them reached their preoperative sports level. Eight patients reported on performing sports “once a week”, 11 patients “two to three times per week” and 9 patients reported on performing sports “more than 3 times a week”. These numbers did not alter statistically significant.

Overall, patients reported on a good subjective functional outcome using the MKQ 65.3% ± 17.4% [22–25]. Jang et al. presented a high Lysholm Score (mean of 89.5) using hook plating for patella fractures which confirms our findings [26]. The range of motion in the presented cohort was rather low (89.1° ± 17.4°; range: 60–120°) compared to current findings by Yao et al. who reported on 124.28° ± 5.09° 6 months postoperatively [27] and other authors [28, 29]. However, the functional outcome of patients was good to excellent from a subjective point of view which is interesting since the restricted range of motion compared to other findings did not significantly decrease the return to sports.

Furthermore, the results showed no statistically significant difference in functional outcome concerning age (*p* = 0.4), body mass index (*p* = 0.6) or fracture pattern (*p* = 0.2). Nevertheless, the functional outcome in patients suffering from OTA C3-type fracture compared to OTA C1-type fractures were perceptibly less favorable yet without statistical significance. Yao et al. recently reported on good-to-excellent results after operative treatment of C2 and C3 patella fractures using a new developed “Ti–Ni shape-memory patella concentrator” [27]. These findings are promising yet controversial since comminuted fractures are prone to less favorable functional outcomes according to the current literature [30, 31].

The detected complication rate in the presented cohort was 5.1% (*n* = 2) which is analogous to prospective findings from Wild et al. in 2016 who reported on two cases which were in need of revision surgery [32].

Furthermore, patients were asked for the presence of AKP (53.6%) and knee joint swelling (35.7%) during sports.

Lazaro et al. postulated a clinical improvement after the first six months after ORIF of patella fractures, yet with persisting functional impairment for 12 months [12]. Besides RTS and activity level, the evaluation focused on persistent complaints. Anterior knee pain (64.3%) was reported “occasionally” and on a “regular basis” by the majority of our patients. This is supported by findings from Lazaro et al. who reported AKP (80%) in patients who were treated using tension-band wiring [12]. A correlation to AKP due to different implants cannot be distinguished at this point. Singh et al. reported on AKP in 10% of their patients (*n* = 20) treated with angle-stable plate fixation in displaced patella fractures [33]. Postoperative findings from Greenberg et al. in 2018 showed no significant increase of function (Kujala and Lysholm Score) after implant removal after ORIF of patella fractures [14]. Our findings are somewhat limited since none of the 39 presented patients required implant removal.

In 2019, Ellwein et al. presented a prospective case series with gradual decrease of complaints of AKP in patella fractures operated with fixed-angle plates [34]. This study does not provide results about different techniques in their functional outcome due to lower absolute numbers. Recently published data by Tengler et al. presented a relatively low complication rate (*n* = 5, 13%) and good clinical outcomes (Lysholm Score: 95 points, Kujala Score: 95 points) in a case series of 38 patients after anterior locking plating of the patella [35]. Additionally, Buschbeck et al. reported on similar results of functional outcome parameters in their retrospective study with 29 patients and locking plate fixation of comminuted patella fractures [30].

LeBrun et al. showed a persistence of significant symptomatic complaints and functional deficits at a mean of 6.5 years after operative treatment of dislocated patella fractures in a prospective cohort study [13]. However, they did not report the functional outcome and fracture severity.

Despite the current literature shows a trend towards fixed-angle plate stabilization, this finding could not be observed in the observational study of 3194 patella fractures from the Swedish Fracture Register in 2022 by Kruse et al. [36]. They reported on 67% of conservatively treated patients and in case of operative treatment, the most used technique was tension-band wiring with 24%.

## Limitations

Due to the retrospective study design, a selection bias was accepted. The low patient numbers can be explained by the strict exclusion criteria to create the most homogenous cohort. The gathered patient-related outcome measurements results inherit a selective reporting bias by the patients. The age distribution of this cohort may not reflect the RTS rate of younger patients and their higher potential and assumable faster rehabilitation. A comparison of different surgical techniques and their functional outcome was too low to achieve statistically reliable results.

## Conclusion

The results of this study demonstrate a high return to sports rate of > 90% after ORIF of patella fractures. However, only 51.6% were able to return to their pre-injury sports level. Neither the type of fracture, nor age or BMI did significantly alter the functional outcome after ORIF of the patella.

## Data Availability

Data and materials are available on reasonable request by the last author.

## Abbreviations

AKP: Anterior knee pain
OTA: Orthopaedic trauma association
BMI: Body mass index
IKDC: International knee documentation committee
IRB: Institutional review board
KOOS: Knee injury and osteoarthritis outcome score
MKQ: Munich knee questionnaire
NRS: Numeric rating scale
NSAID: Non-steroidal anti-inflammatory drug
ORIF: Open-reduction internal-fixation
RTS: Return to sports
*ns: Not significant
